# Focus on endeavor for creation of materials–tissues intelligent interface

**DOI:** 10.1080/14686996.2017.1354482

**Published:** 2017-07-27

**Authors:** Takao Hanawa

**Affiliations:** ^a^ Institute of Biomaterials and Bioengineering, Tokyo Medical and Dental University, Tokyo, Japan

When a material is implanted into a human body, an immediate reaction occurs between its surface and the living tissues. In other words, an immediate reaction at this initial stage determines and defines the material–tissue compatibility. A synthetic material usually creates a clear interface with a biological system, which may include cells, bacteria, and tissues, and this interface works as a barrier for transportation of molecules and execution of biofunctions. On the other hand, if we could create a gradual, seamless interface allowing for a smooth transport of molecules, both material and tissue would be integrated together. This structure may be defined as biosis–abiosis intelligent interface. It is expected to support not only chemical biofunctional conduction, but also mechanical stress conduction. But how can we create such a biosis–abiosis intelligent interface? In addition, tissue formation on materials and tissue adhesion to the materials are conventionally discussed without any bacterial adhesion and associated infection. Water molecules, inorganic ions, and proteins adhere to the material surface, which is followed by adhesion of cells and finally the tissue formation. Therefore, this interface contains a space and time hierarchy. Moreover, the topic is related to bio-sensing and nano-medicine. This Focus Issue is devoted to solving the problems described above. It will hopefully improve our understanding of the current and future research trends in the interface between human tissues and materials.

